# Towards understanding partial adaptation to the subterranean habitat in the European cave spider, *Meta menardi*: An ecocytological approach

**DOI:** 10.1038/s41598-019-45291-z

**Published:** 2019-06-24

**Authors:** Saška Lipovšek, Gerd Leitinger, Franc Janžekovič, Peter Kozel, Barbara Dariš, Matjaž Perc, Dušan Devetak, Nina Weiland, Tone Novak

**Affiliations:** 1 Faculty of Medicine, University of Maribor, Taborska ulica 8, 2000 Maribor, Slovenia; 2 Department of Biology, Faculty of Natural Sciences and Mathematics, University of Maribor, Koroška cesta 160, 2000 Maribor, Slovenia; 3 Faculty of Chemistry and Chemical Engineering, Smetanova ulica 17, University of Maribor, 2000 Maribor, Slovenia; 4 Gottfried Schatz Research Center, Division of Cell Biology, Histology and Embryology, Medical University of Graz, Neue Stiftingtalstrasse 6, 8010 Graz, Austria; 5 Karst Research Institute ZRC SAZU, Titov trg 2, 6230 Postojna, Slovenia; 6 UNESCO Chair on Karst Education, University of Nova Gorica, Glavni trg 8, 5271 Vipava, Slovenia; 7 Department of Physics, Faculty of Natural Sciences and Mathematics, University of Maribor, Koroška cesta 160, 2000 Maribor, Slovenia; 8 Vodovodna ulica 27, 2352 Selnica ob Dravi, Slovenia

**Keywords:** Ecosystem ecology, Cellular imaging

## Abstract

The European cave spider, *Meta menardi*, is a representative of the troglophiles, i.e. non-strictly subterranean organisms. Our aim was to interpret the cytological results from an ecological perspective, and provide a synthesis of the hitherto knowledge about *M. menardi* into a theory of key features marking it a troglophile. We studied ultrastructural changes of the midgut epithelial cells in individuals spending winter under natural conditions in caves, using light microscopy and TEM. The midgut diverticula epithelium consisted of secretory cells, digestive cells and adipocytes. During winter, gradual vacuolization of some digestive cells appeared, and some necrotic digestive cells and necrotic adipocytes appeared. This cytological information completes previous studies on *M. menardi* starved under controlled conditions in the laboratory. In experimental starvation and natural winter conditions, *M. menardi* gradually exploit reserve compounds from spherites, protein granules and through autophagy, and energy-supplying lipids and glycogen, as do many overwintering arthropods. We found no special cellular response to living in the habitat. Features that make it partly adapted to the subterranean habitat include starvation hardiness as a possible preadaptation, an extremely opportunistic diet, a partly reduced orb, tracking and capturing prey on bare walls and partly reduced tolerance to below-zero temperatures.

## Introduction

The European cave spider, *Meta menardi* (Latreille, 1804) (Araneae, Tetragnathidae), is a ubiquitous species inhabiting the twilight zone of many hypogean habitats across Europe^1–7^. With an adult body size of 10 to 17 mm, *M. menardi* is among the most noticeable animals of the entrance cave zone^1–3,5,6,8–20^. The life cycle involves two ecophases, a hypogean and an epigean one^5^. In spring, adults mate in hypogean habitats, like caves, where in summer females produce egg sacs (cocoons). Juveniles hatch in the late autumn or in winter, but remain within the egg sacs until early spring, when the second-instar spiderlings leave the caves and spread outside by ballooning. They live in epigean habitats until becoming fourth-stage instars, which return to the hypogean habitats^3,5,16^. *Meta menardi* is mainly a sit-and-wait predator^21^, building a relatively small, planar orb-web with an open hub, but with a mesh size (length of the individual sticky spiral sections between adjacent radii) almost twice the size of other comparably large orb weavers. Such an orb does not ensnare small prey; this is compensated by occasional leaving the orb to track and capture prey in the vicinity^1,12,15,21–24^. Many other authors have made important contributions to the biology and ecology of *M. menardi*^22,25–34^.

According to the general ecological classification of subterranean animals^35–37^, trogloxenes are species regularly found in the subterranean habitat, but unable to complete their entire life cycles therein, and troglobionts are those regularly found in the subterranean habitat and completing their entire life cycles therein^37^. *Meta menardi* ranks among the troglophiles^38^, which are in between. These species complete their life cycles either in the subterranean or in the surface environments, forming populations in both habitats^37,39^. Most of them show some moderate adaptation to the subterranean environment, such as partly reduced eyes and adaptations to compensate for the lack of visual orientation^39,40^. They have partly reduced tolerance to below-zero temperatures^41,42^. The attainment of mechanisms that may be more efficient in regulation of hydric balance and metabolism, combined with the ability to carry out the entire life cycle in darkness, implies complete adaptation to subterranean conditions^7^. Showing partial adaptation to the subterranean habitat, troglophiles may provide insight into possible adaptatiogenesis to this habitat; *M. menardi* could well serve as a model species in this respect.

*Meta menardi* are active throughout the year^5,34^, and in caves they do not spend the winter in dormancy; they feed occasionally when prey is available^43^. In central Europe, during winter, two groups of potential prey are present. The first group consists of about 50 species that are in low abundances present in the cave all over the year. Additionally, the second group of about 20 overwintering species enter caves during the late fall and leave them in spring^2^. Individuals of this group are available only during migrations, since the spiders do not detect resting prey.

In our previous research into understanding the survival strategy of *M. menardi* during times of prey deficiency, we studied ultrastructural changes of the midgut epithelial cells under controlled starvation conditions^33,43^. We chose the midgut epithelium cells, since these show rapid response to the feeding conditions of an individual^44^. We carried out these experiments during the growing period−in spring and in autumn−with relatively abundant prey in the entrance cave sections^33^, and in winter, when prey is usually scarce^43^. We found that during the growth period, *M. menardi* accumulate reserve compounds in spherites and protein granules, and energy-supplying lipids and glycogen, all of which form an adaptive response to potential starvation^43^. This response is, in general, the same as in invertebrates with winter dormancy in their life cycle, e.g. *Scoliopteryx libatrix*^45^.

The midgut epithelium of spiders consists, in general, of four cell types: basal, secretory and digestive cells and guanocytes^46,47^. In *M. menardi*, secretory and digestive cells and adipocytes are present^33,43^. In starved *M. menardi*, macroautophagy−referred to as autophagy^48,49^−is an indicative pro-survival process^33,50^.

In cases when *M. menardi* do not catch prey in winter, they undergo a kind of natural starvation similar to winter dormancy in other invertebrates (e.g.^33,51–55^). We use the word “wintering” here (“overwintering” in our previous studies^33,43^) to designate conditions in *M. menardi* in winter. We hypothesized that some food is available to *M. menardi* during winter in caves, but it is too scarce to prevent the ultrastructural changes characteristic of spiders starved under controlled conditions. Moreover, we assumed that in starved *M. menardi*, the same types of changes as in other naturally starved arthropods during overwintering would appear in cells.

We asked the following questions. (1) What changes appear in the midgut diverticula epithelial cells? And which energy-supplying compounds do *M. menardi* spend while wintering in natural conditions? (2) Are there any differences in this respect between experimental starvation under controlled and starvation under natural conditions in caves? (3) How can this knowledge contribute to understanding adaptatiogenesis in spiders to the subterranean habitat? Finally, we merged relevant hitherto knowledge and established the theory on the nature of the adaptation of *M. menardi* being intermediate between the epigean and the deep subterranean spider species.

## Results

**Figure 1:**
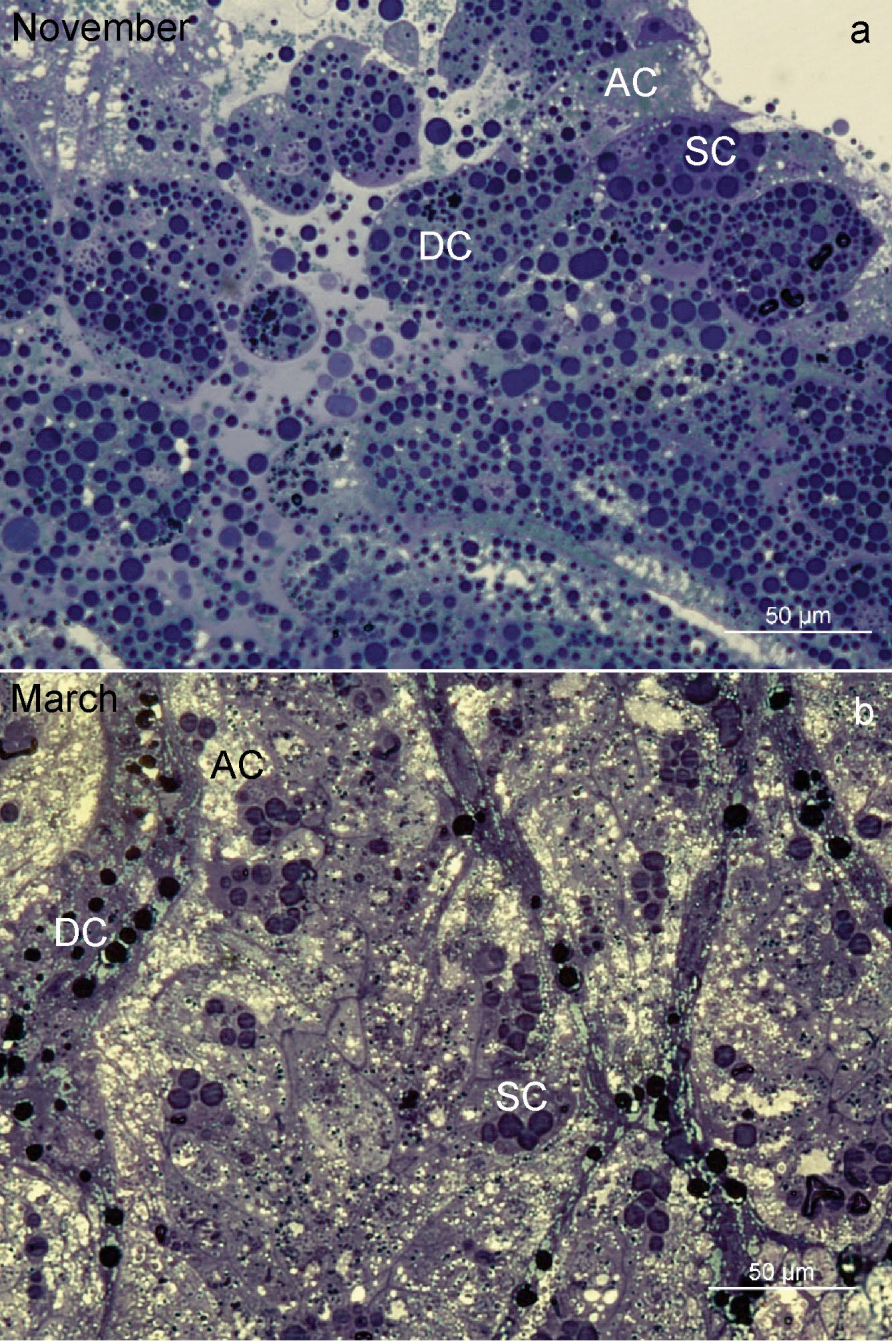
Semithin section of the midgut diverticula of *Meta menardi*. (**a**) The beginning of overwintering (November). (**b**) The end of overwintering (March). AC, adipocyte; DC, digestive cell; SC, secretory cell.

In both sexes, during wintering, the midgut was composed of a branched system of diverticula, with the epithelium composed of digestive cells, secretory cells and adipocytes (Fig. 1). The structure of the midgut diverticula epithelial cells changed during wintering. The most characteristic structural change was a progressive vacuolization of all three cell types (Fig. 1a,b).

### Secretory cells

**Figure 2:**
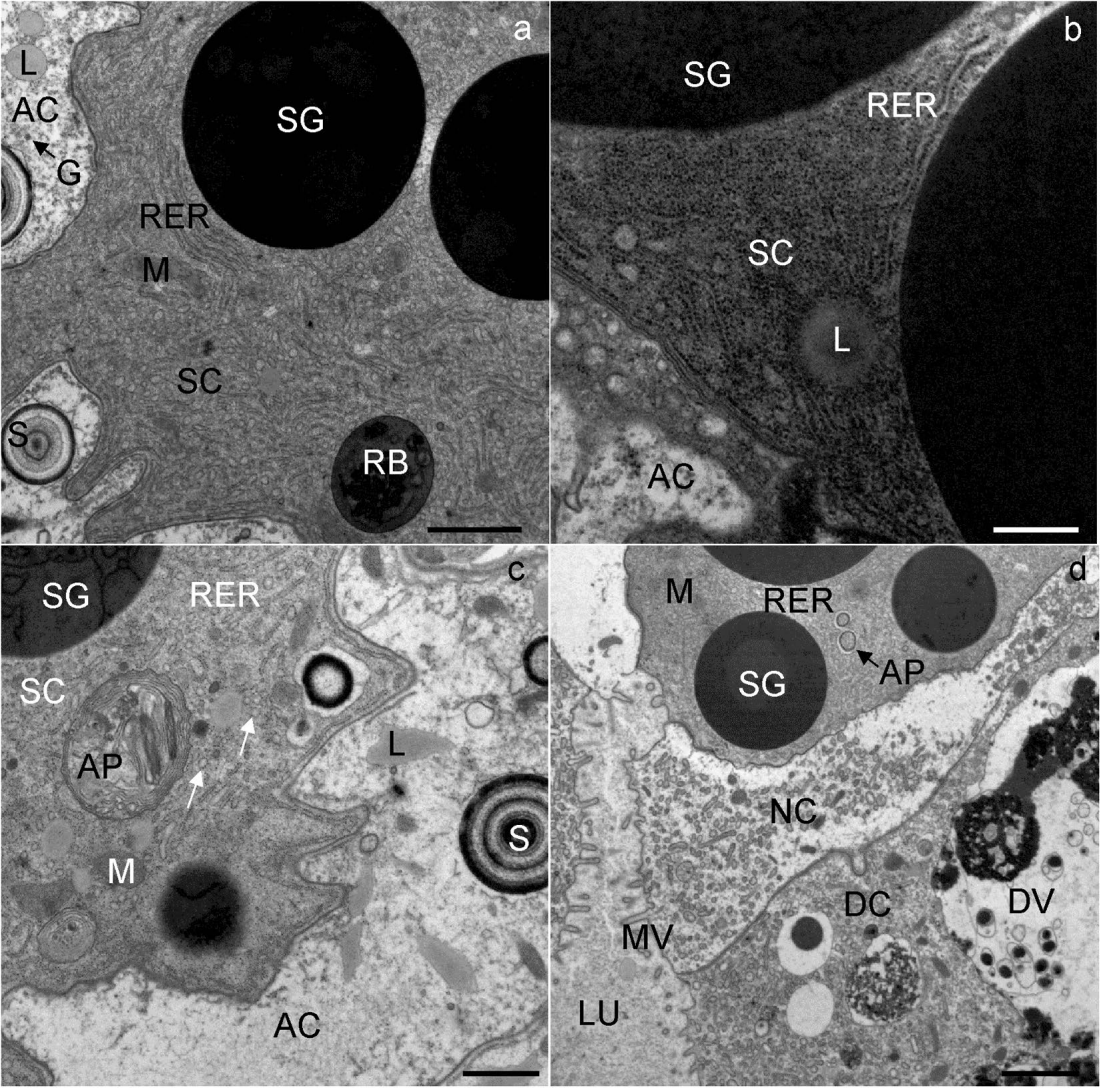
Ultrathin section of the secretory cells in the midgut diverticula of *M. menardi*. The beginning of overwintering in November; (**a**) male); (**b**) female. (**c**) The middle of overwintering in January (female). (**d**) The end of overwintering in March (male). AC, adipocyte; AP, autophagosome; DC, digestive cell; DV, digestive vacuole; G, glycogen rosettes; L, lipid droplet; LU, lumen of the midgut; M, mitochondrium; MV, microvilli; NC, necrotic cell; RER, rough endoplasmic reticulum; RB, residual body; S, spherite; SC, secretory cell; SG, secretory granulum; arrows indicate small vacuoles in the cytoplasm. Scale bars: (**a**) 2 µm; (**b**) 500 nm; (**c**) 1 µm; (**d**) 2 µm.

At the beginning of wintering, the secretory cells contained an abundant rough endoplasmic reticulum (RER), many electron-dense secretory granules (Fig. 2a,b), mitochondria, spherites (Fig. 2a) and Golgi complexes. In some secretory cells, a few lipid droplets were seen (Fig. 2b). A round to oval nucleus was located centrally in the cell.

In the middle and at the end of wintering, the general structure of the secretory cells was comparable to that at the beginning of wintering; the only remarkable difference was the presence of individual autophagic structures in some secretory cells. Autophagosomes (Fig. 2c,d) and autolysosomes were the most frequent autophagic structures. In the cytoplasm of some secretory cells, a few vacuoles were present (Fig. 2c).

### Digestive cells

**Figure 3:**
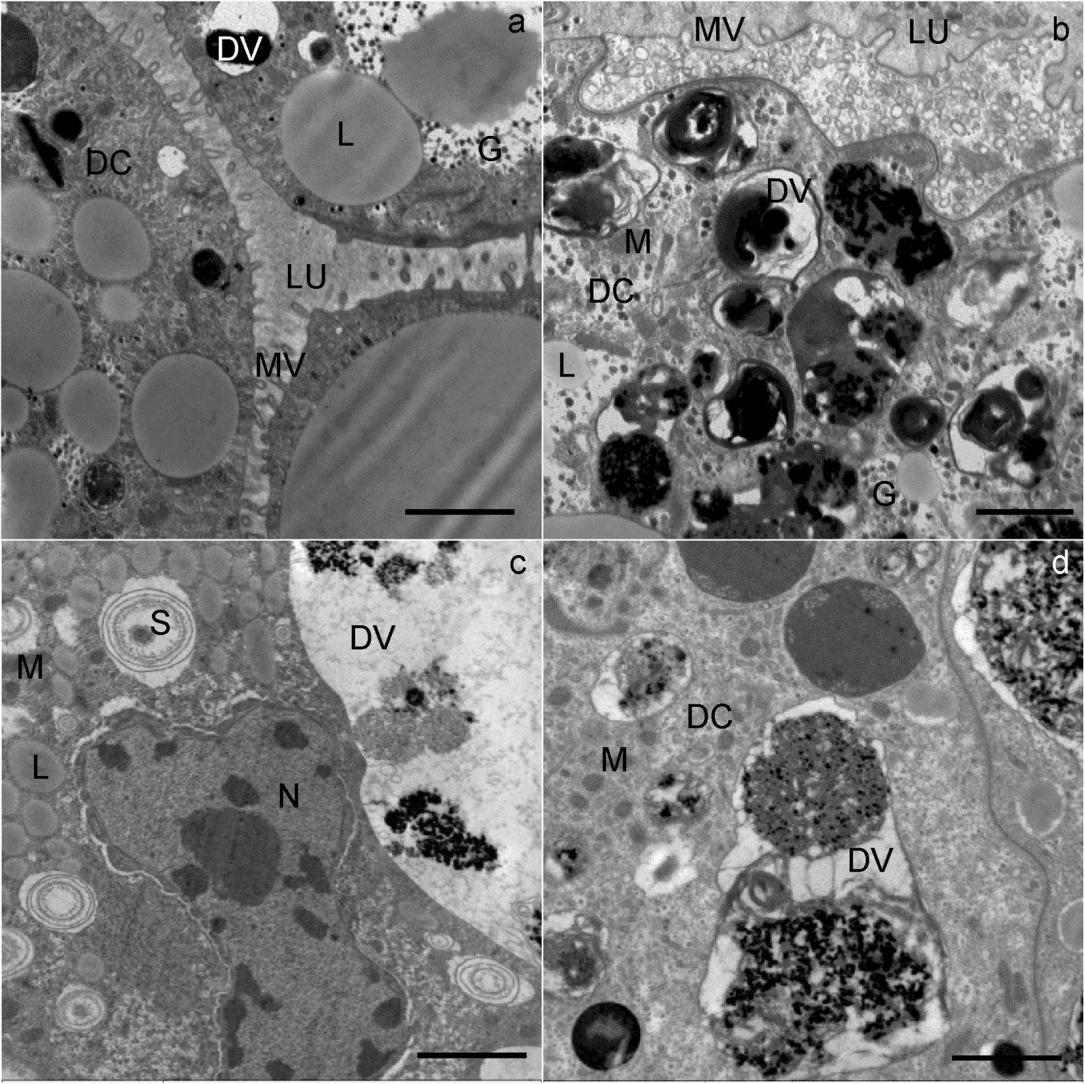
Ultrathin section of the digestive cells in the midgut diverticula of *M. menardi*. The beginning of overwintering in November; (**a**) male; (**b**) female. The middle of overwintering in January; (**c**) male); (**d**) female. DC, digestive cell; DV, digestive vacuole; G, glycogen rosettes; L, lipid droplet; LU, lumen of the midgut; M, mitochondrium; MV, microvilli; N, nucleus; S, spherite. Scale bars: (**a**) 1 µm; (**b–d**) 2 µm.

At the beginning of wintering, the apical plasma membrane of the digestive cells was differentiated into numerous microvilli projecting into the lumen of the midgut diverticulum (Fig. 3a). The digestive cells were characterized by digestive vacuoles, located predominantly in the apical part of the cell, and containing material of different electron density (Fig. 3b). Besides the digestive vacuoles, the cytoplasm contained lipid droplets (Fig. 3a), mitochondria, spherites, a rough endoplasmic reticulum and Golgi apparatus. The spherites were round, composed mostly of concentric layers of electron-lucent and electron-dense material, and a membrane. A round to oval nucleus was located centrally in the cell.

**Figure 4:**
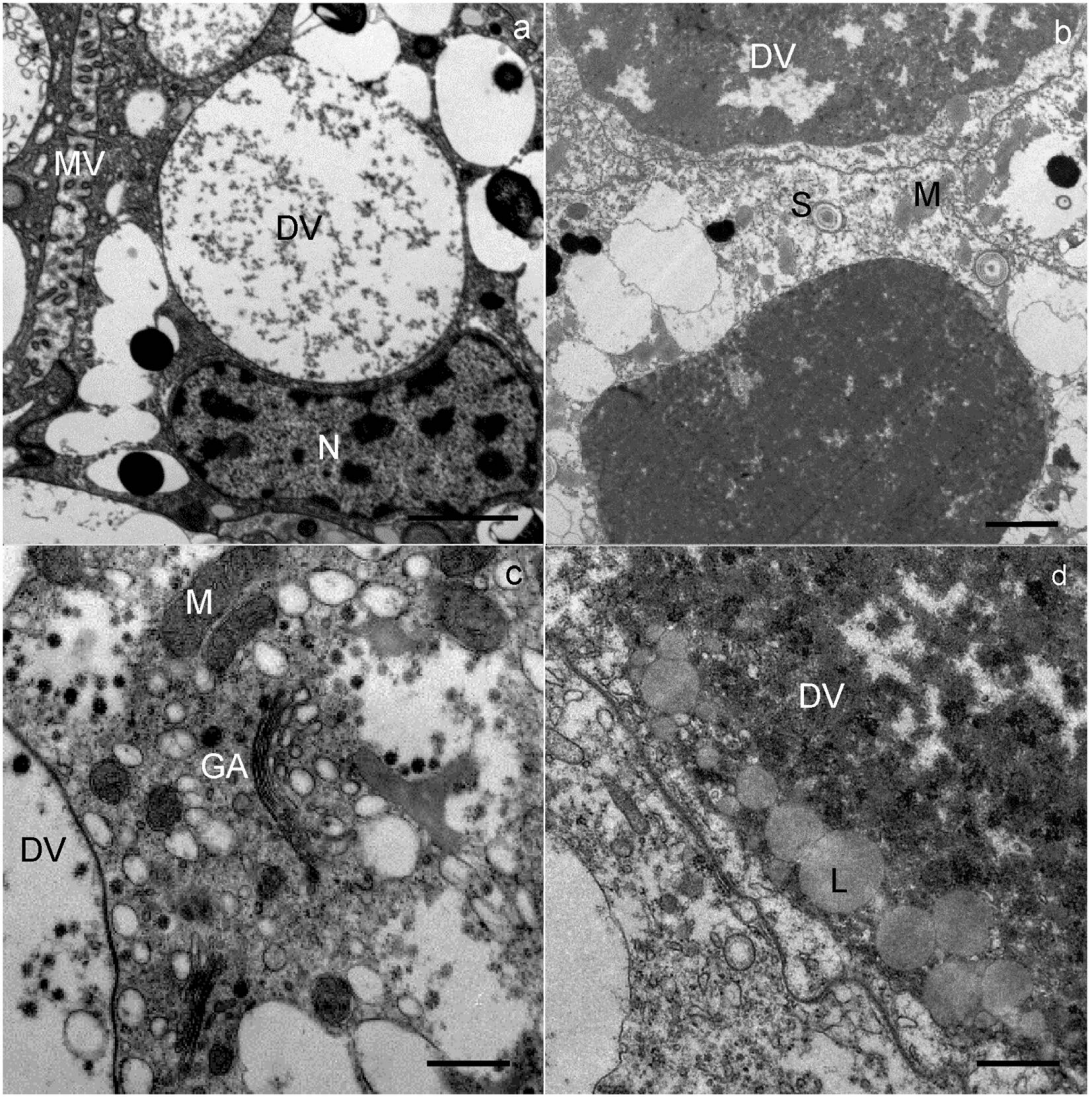
Ultrathin section of the digestive cells of the midgut diverticula of *M. menardi* at the end of overwintering in March; (**a**) male; (**b–d**) female. DV, digestive vacuole; GA, Golgi apparatus; L, lipid droplet; M, mitochondrium; MV, microvilli; N, nucleus. Scale bars: (**a**) 1 µm; (**b,c**) 2 µm; (**d**) 500 nm.

In the middle and at the end of wintering, the general structure of the digestive cells was comparable to that of cells at the beginning of wintering. In the middle and at the end of wintering, the epithelium of the midgut diverticula contained some necrotic digestive cells (Fig. 2d) and numerous autophagic structures, mostly autophagosomes and autolysosomes. In the middle of wintering, in most digestive cells there was a large central digestive vacuole containing only remnants of material of different electron densities (Fig. 3c). Additionally, a few digestive cells contained smaller, peripheral digestive vacuoles with electron-dense material (Fig. 3d). Spherites consisted of a few electron-dense concentric layers (Fig. 3c). At the end of wintering, almost all digestive cells contained a single large digestive vacuole with a homogeneous fluid or with a flocculent material (Fig. 4a). The cytoplasm of many digestive cells was vacuolised (Fig. 4c). In many digestive cells, the Golgi apparatus could clearly be seen (Fig. 4c).

However, in one female, the ultrastucture of the digestive cells differed conspicuously from those in the other specimens by containing a large digestive vacuole, filled with electron-dense material (Fig. 4b). In the periphery of some digestive vacuoles, small lipid droplets were seen (Fig. 4d). These digestive cells were of typical appearance, as in well-fed individuals, meaning that this female had fed in winter in the cave just a few hours before being picked up for the study.

### Adipocytes

**Figure 5:**
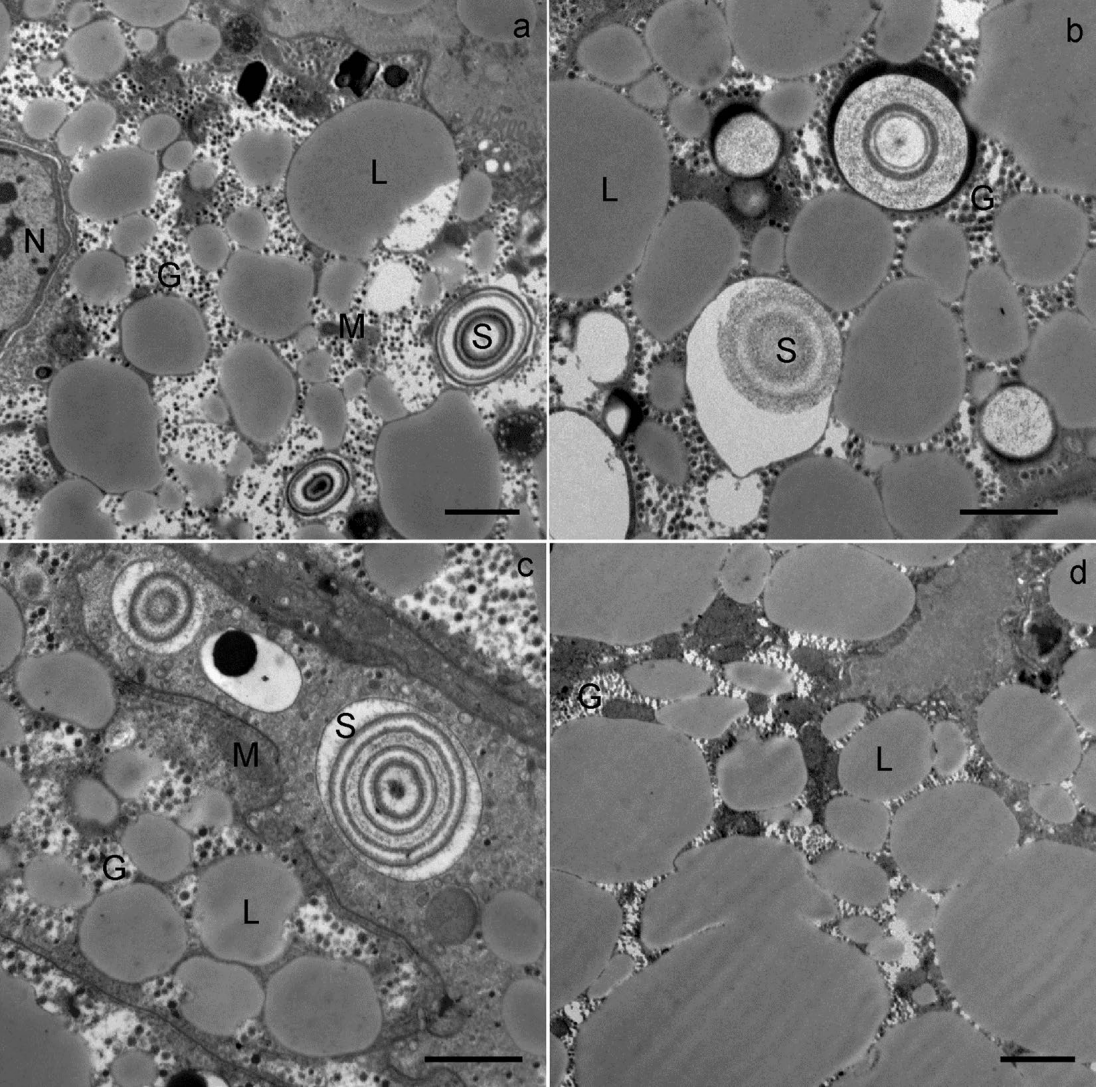
Ultrathin section of the adipocytes in the midgut diverticula of *M. menardi* at the beginning of overwintering in November; (**a–c**) male; (**d**) female. G, glycogen rosettes; L, lipid droplet; M, mitochondrium; N, nucleus; S, spherite. Scale bars: (**a,d**) 2 µm; (**b,c**) 1 µm.

**Figure 6:**
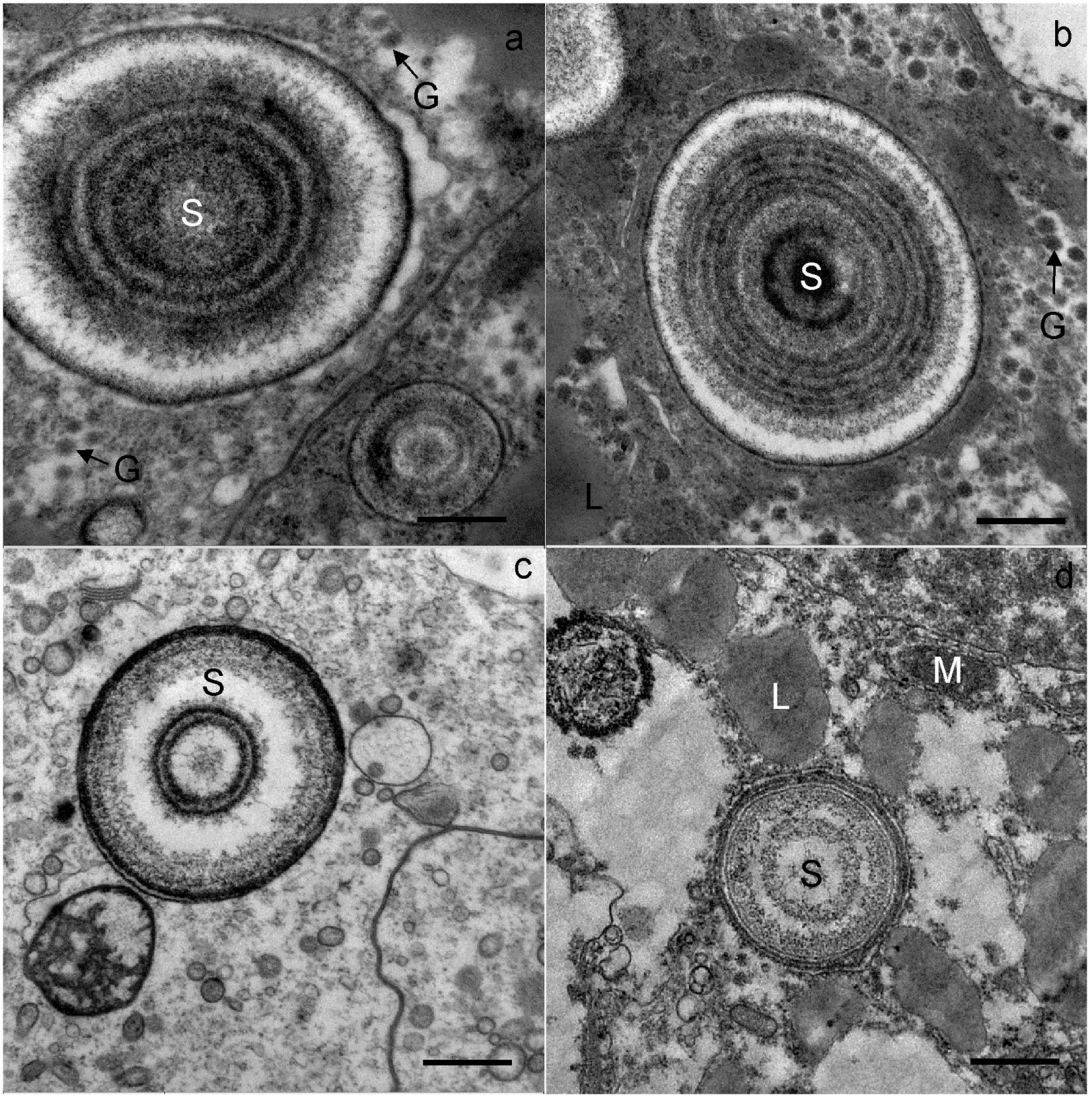
Ultrathin section of the adipocytes in the midgut diverticula of *M. menardi*, showing spherites. The beginning of overwintering in November; (**a)** male; (**b**) female. The middle of overwintering in January; (**c**) male; (**d**) female. G, glycogen rosettes; L, lipid droplet; S, spherite. Scale bars: 500 nm.

**Figure 7:**
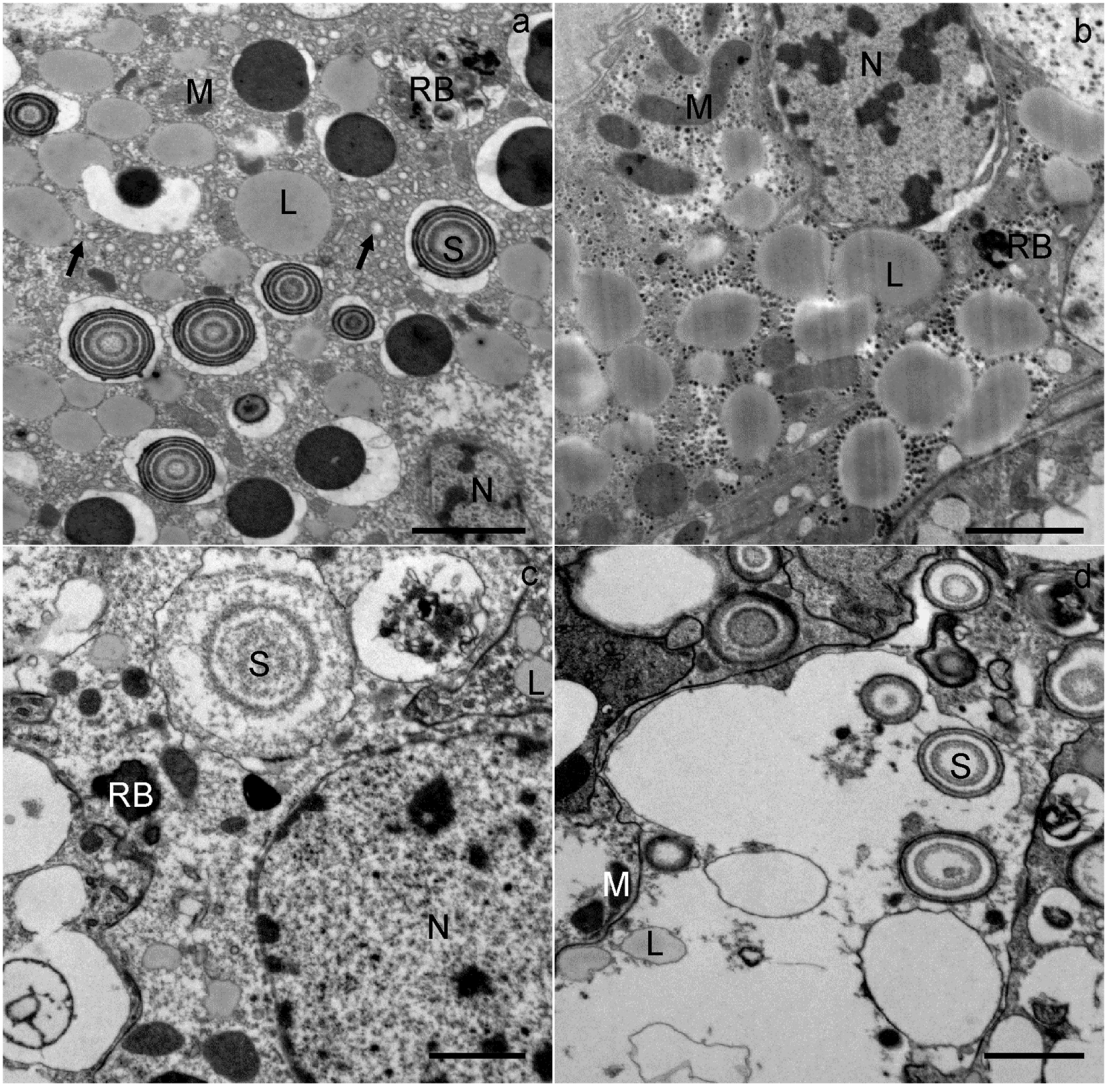
Ultrathin section of the adipocytes in the midgut diverticula of *M. menardi*. The middle of overwintering in January; (**a**) male; (**b**) female. The end of overwintering in March; (**c**) male; (**d**) female. L, lipid droplet; M, mitochondrium; N, nucleus; RB, residual body. The arrows show vacuoles in the cytoplasm. Scale bars: 2 µm.

At the beginning of wintering, the cytoplasm of the adipocytes contained numerous lipid droplets, glycogen rosettes and spherites, with concentric layers of electron-lucent and electron-dense material (Figs 5a–c and 6a,b). Nuclei were oval or irregularly shaped because of the pressure of many lipid droplets. In the middle and at the end of wintering, the cytoplasm was vacuolised (Fig. 7a). The reserve compounds were reduced (Fig. 7a,b), while the autophagic structures were more numerous as compared with individuals at the beginning of wintering. Autophagosomes and residual bodies (Fig. 7b,c) predominated. Most spherites showed structural changes in comparison to those at the beginning of the wintering; either they were composed of a few concentric layers of exclusively electron-dense material and a spherital membrane (Fig. 6c,d), or the material of some spherites was completely exploited, with only the membrane being preserved (Fig. 7d). In some adipocytes, the material of the spherites accumulated in one larger vacuole (Fig. 7d).

### Quantification of autophagic structures

**Figure 8:**
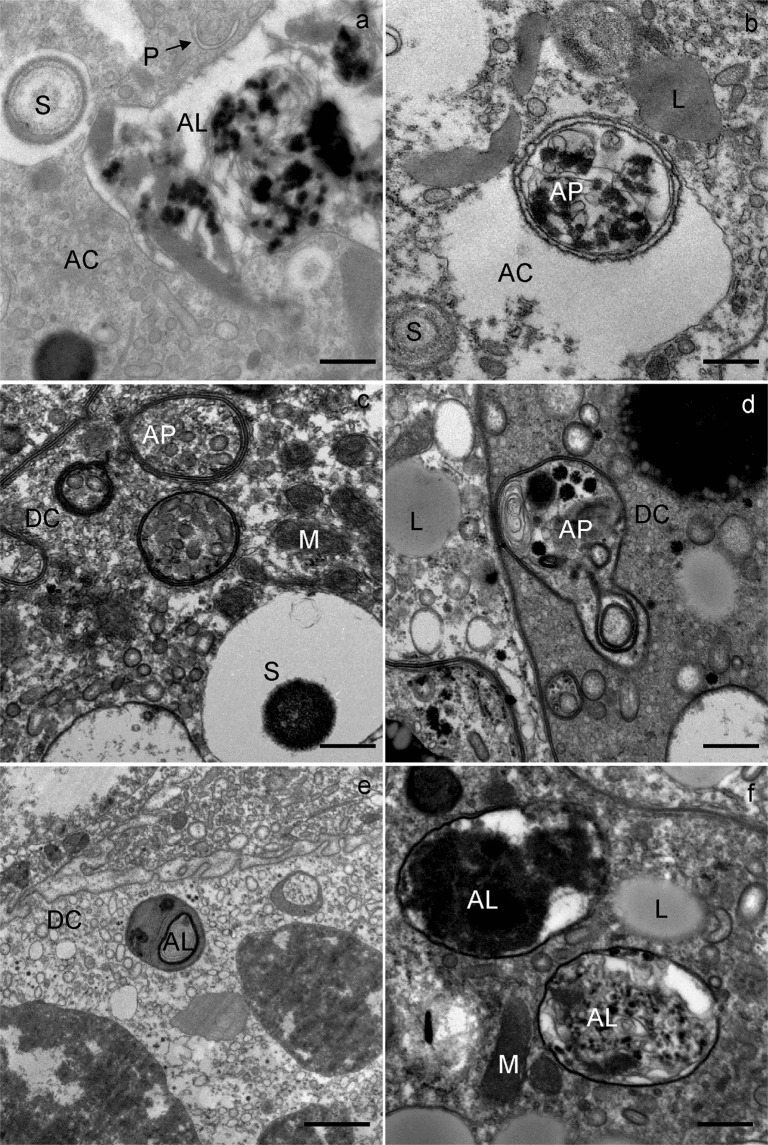
Ultrathin section of the midgut diverticula epithelial cells of *M. menardi*, showing autophagic structures. The middle of overwintering in January; (**a**) male; (**b**) female. The end of overwintering in March; (**c**) male; (**d–f**) female. AC, adipocyte; AL, autolysosome; AP, autophagosome; DC, digestive cell; G, glycogen rosettes; L, lipid droplet; M, mitochondrium; P, phagophore; RB, residual body; S, spherite. Scale bars: (**a–d**,**f**) 500 nm; (**e**) 1 µm.

Phagophores (Fig. 8a), autophagosomes (Fig. 8b–d), autolysosomes (Fig. 8e,f) and residual bodies (Figs 2a and 7c) were present in all the three cell types. The percentage rates of autophagic cells increased from the beginning until the end of wintering (Table 1).

**Table 1:** Percentage rates of midgut epithelial cells with autophagic structures in *Meta menardi* during overwintering in natural conditions in caves, as observed by TEM.

Time frame Sex	Beginning	Middle	End
♂	12	42	69
♀	13	45	67

### Quantification of reserve lipids, glycogen and proteins

**Figure 9:**
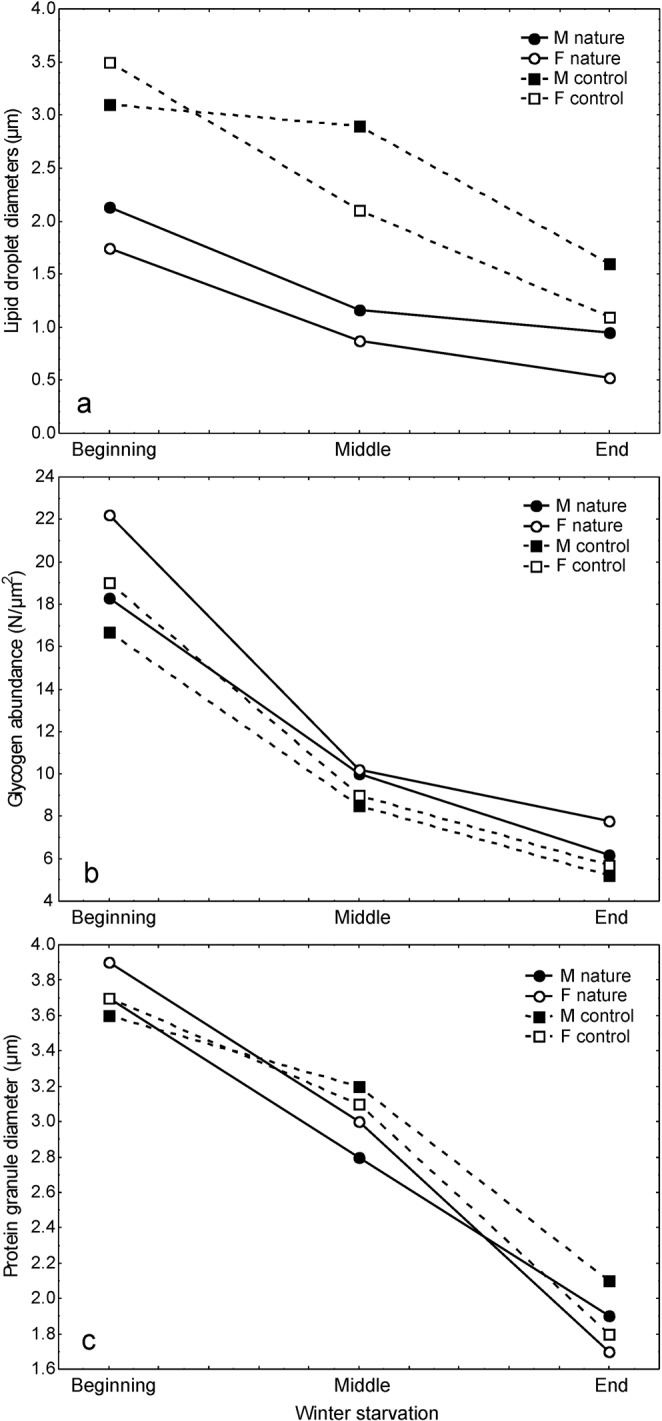
Mean values of (**a**) lipid droplet diameters, (**b**) glycogen rosette abundance and (**c**) protein granule diameter in the midgut epithelial cells of *Meta menardi* during starvation under controlled conditions in the laboratory and in natural conditions in caves.

The descriptive values for lipid droplet diameters, protein granule diameters, and the abundance of glycogen rosettes in the midgut epithelial cells of *M. menardi* during wintering in caves are shown in Table 2. Differences were significant in lipid droplets and protein granule diameters, and glycogen rosette counts among time frame, sex, and a combination of time frame and sex, except for protein granule diameter between sexes (Table 3). In both sexes, the use of lipids, according to lipid droplet diameters, was more intensive in the first half of wintering (Fig. 9a). From the beginning until the middle of wintering, the mean lipid droplet diameter diminished by 0.016 µm/day in males, and by 0.015 µm/day in females, and from the middle until the end of wintering, by 0.003 µm/day, and by 0.006 µm/day, respectively.

**Table 2:** Descriptive statistics for lipid droplet diameter, protein granule diameter and glycogen rosette abundance in the midgut epithelial cells of *Meta menardi* in natural conditions in caves, sample: sex in each time frame.

Sex	Time frame	Lipid droplet diameter (μm) (N = number per sample)	Glycogen rosette abundance (N/μm^2^) (N per sample = 30)	Protein granule diameter (μm) (N per sample = 30)
		Mean ± St.Dev (N) Min–Max	Mean ± St.Dev Min–Max	Mean ± St.Dev Min–Max
♂	Beginning – Nov.	2.1 ± 1.9 (310) 0.3–12.3	18.3 ± 4.28 10–27	3.7 ± 0.5 2.6–4.5
	Middle – Jan.	1.2 ± 0.7 (371) 0.4–8.0	10.0 ± 3.37 4–17	2.8 ± 0.5 2.0–4.1
	End – Mar.	1.0 ± 0.9 (298) 0.1–6.0	6.2 ± 2.16 2–10	1.9 ± 0.4 1.2–2.6
♀	Beginning – Nov.	1.7 ± 1.3 (500) 0.4–11.6	22.2 ± 2.86 17–29	3.9 ± 0.4 2.6–4.7
	Middle – Jan.	0.9 ± 0.5 (602) 0.2–3.2	10.2 ± 3.34 2–17	3.0 ± 0.4 2.2–3.7
	End – Mar.	0.5 ± 0.4 (614) 0.0–2–0	7.8 ± 2.38 3–12	1.7 ± 0.5 0.6–2.6

**Table 3:** Two-way ANOVA of lipid droplet diameter, glycogen rosette abundance and protein granule diameter in the midgut epithelial cells of *Meta menardi* between time frames of overwintering and sexes.

	SS	Df	MS	F	p
**Lipid droplet diameter**					
Intercept	10.1	1	10.1	106.77	**<0.001**
Time frame	98.6	2	49.3	520.34	**<0.001**
Sex	14.0	1	14.0	147.55	**<0.001**
Time frame * Sex	4.9	2	2.5	25.94	**<0.001**
Error	254.7	2689	0.1		
**Glycogen rosette abundance**					
Intercept	27925.4	1	27925.4	2824.35	**<0.001**
Time frame	5773.3	2	2886.7	291.96	**<0.001**
Sex	168.2	1	168.2	17.01	**0.001**
Time frame * Sex	104.7	2	52.4	5.29	**0.006**
Error	1720.4	174	9.9		
**Protein granule diameter**					
Intercept	1428.1	1	1428.1	6784.76	**<0.001**
Time frame	120.3	2	60.1	285.72	**<0.001**
Sex	0.2	1	0.2	1.01	0.315
Time frame * Sex	1.8	2	0.9	4.36	**0.014**
Error	36.6	174	0.2		

Simple and combined parameters are presented. Significant differences in bold.

In both sexes, the exploitation of glycogen was more intensive in the first half of the experiment (Fig. 9b). From the beginning until the middle of wintering, the mean glycogen rosette abundances diminished by 0.14 rosettes/μm^2^/day in males, and by 0.20 rosettes/μm^2^/day in females, and from the middle until the end of wintering by 0.06 and by 0.04 rosettes/μm^2^/day, respectively.

In both sexes, the exploitation of proteins was steady during wintering (Fig. 9c). From the beginning until the middle of wintering, the mean protein granule diameter diminished by 0.015 µm/day in both sexes, and from the middle until the end of wintering, by 0.015 µm/day in males, and by 0.022 µm/day in females.

Table 4 shows the differences in lipid droplet diameter, protein granule diameter and glycogen rosette abundances in the midgut epithelial cells of *Meta menardi* in winter, undergoing starvation under controlled and under natural conditions in caves.

**Table 4:** Testing differences in lipid droplet diameter, protein granule diameter and glycogen granule counts in the midgut epithelial cells of *Meta menardi* in winter undergoing starvation under controlled conditions in the laboratory and natural conditions in caves.

Sex	Time frame of starvation	Lipid droplet diameter	Glycogen rosette counts	Protein granule diameter
		t test p	t test p	t test p
♂	Beginning – Nov.	**4.47** < **0.001**	1.62 0.110	1.06 0.296
	Middle – Jan.	**16,28** < **0.001**	**2.08** **0.042**	**2.33** **0.023**
	End – Mar.	**5.86** < **0.001**	**2.01** **0.049**	1.66 0.102
♀	Beginning – Nov.	**11.52** < **0.001**	**4.51** < **0.001**	1.72 0.092
	Middle – Jan.	**20.26** < **0.001**	1.54 0.1292	0.91 0.366
	End – Mar.	**11.97** < **0.001**	**3.91** **0.001**	0.92 0.361

## Discussion

Many subterranean spiders have evolved special foraging behaviours and feeding habits, in order to accommodate the generally low availability of prey^7^. In *M. menardi*, the course of starvation processes provides insight into adaptation to the subterranean habitat at the cellular level. In our previous research, we studied the ultrastructural changes in the midgut diverticula epithelial cells of *M. menardi* that had been starved under controlled conditions in the growth period^33^ and in winter^43^. In the present study, we investigated the ultrastructural changes in these cells of *M. menardi* wintering under natural conditions in caves to compare the results with those from experimental starvation in winter under controlled conditions^43^, and to draw overall conclusions.

Most spiders in the temperate zone winter in a rigid posture, especially in the litter, which protects them against extreme temperatures and desiccation^46,56^. In contrast, we confirmed that *M. menardi* do feed in caves in winter if prey is available. Thus, in this respect, *M. menardi* opportunistically feed all through the year, with no special adaptation in the trophic niche to the subterranean habitat, and differs from spiders overwintering in torpor mainly in their temporal niche, and in its extreme opportunistic preying, even including gastropods in their diet^2,30^. *Meta menardi* is ranked among the troglophiles because the individuals dwell in the twilight cave zone with temperatures above the freezing point^5,34,57^. In this sense, the spatial niche of *M. menardi* refers to a stenoecious restriction to subterranean habitats with such conditions, which allow them to stay active throughout the year. In the light of the source−sink model^37,58,59^, *M. menardi* assumingly evolved from the epigean ancestors, forming the epigean source populations through the epigean sink to the recent hypogean source populations. We speculate that the epigean, dispersal ecophase, comprising exclusively young juveniles, possibly corresponds to a residue of a precursory epigean sink population.

The midgut of *M. menardi* consists of a branched system of diverticula, as in other spiders^47,60^ and harvestmen^61^. With the exception of one female, which had fed just before being collected for the study, in *M. menardi* wintering in caves under natural conditions, the ultrastructure of the midgut epithelial cells–digestive cells, secretory cells and adipocytes–did not differ from the ultrastructure during experimental winter starvation^43^. At the beginning of wintering in natural condition in caves, all the epithelial cells were of normal appearance and crowded with reserve substances, revealing that the examined individuals were well fed. Changes in the ultrastructure of the midgut epithelium cells during wintering in caves were generally identical to those in experimentally starved individuals in winter^43^. In the middle and at the end of wintering in caves, vacuolised cytoplasm was characteristic of many midgut epithelial cells. A few necrotic digestive cells were seen in the middle and at the end of wintering. These cells were electron-lucent and contained remnants of decomposed organelles. In the middle and at the end of natural wintering, the midgut epithelial cells were characterized by phagophores, autophagosomes, autolysosomes and residual bodies, as in the experimental conditions^43^. Autophagy, which supports the survival of starving cells, proved to be an important adaptation process in arthropods, e.g. in the overwintering harvestmen *Gyas annulatus*^62^ and *Amilenus aurantiacus*^52^, and in *M. menardi* during experimental starvation^43^. In *M. menardi* wintering in caves, the autophagic structures were often seen in digestive cells and adipocytes, but rarely in secretory cells.

Spherites support the vital cell processes during starvation. At the beginning of wintering in caves, the spherites were round, and composed of concentric, electron-lucent and electron-dense layers and a membrane. By the middle and at the end of wintering, the material of some spherites was partly or completely exploited. In some cells, the exploited spherites accumulated in one larger vacuole. Similar changes were found in the midgut epithelial cells in harvestmen *Gyas annulatus*^62^ and *Amilenus aurantiacus*^52^ and the dipluran *Campodea* (*Monocampa*) *quilisi*^63^. Structural changes of spherites in *M. menardi* wintering in experimental conditions in spring and autumn^33^ and in winter^43^, and under natural conditions in winter (this study) were quite comparable.

As in other arthropods^52,64–66^, in winter starvation under controlled conditions^43^ and in *M. menardi* wintering in natural conditions in caves, lipid, glycogen and protein reserves were gradually depleted from the beginning until the end of the study period. The amounts of reserve lipid, glycogen and protein in *M. menardi* in caves in winter differed considerably from the levels in those under controlled conditions (Table 4), while the patterns for exhausting all three reserve compounds were quite similar (Fig. 9). Although the *M. menardi* individuals being studied during winter starvation under controlled and natural conditions were collected in the same caves on the same dates, those selected for holding in captivity were better fed (Fig. 9a), by chance. This resulted in larger lipid reserves in the cells of the experimental group. In contrast, the amount of glycogen rosettes and the protein granule diameter differed negligibly between the two groups. This is because lipids are the first-level energy reserve compounds in *M. menardi* depending strictly on available prey. Such an event was well documented in the female wintering in the cave, which had fed a few hours before the analysis: In accordance with the midgut diverticula role of absorption, synthesis and storage of lipids, and the transfer of energy supplying compounds^67^, numerous newly emerged lipid droplets were present in the digestive cells. On the other hand, the quite comparable courses of depletion among all three reserve compounds was a consequence of the fact, as explained for insects^63^, that organisms need to expend energy constantly, and if they are not feeding, they must live on reserves accumulated in periods of food abundance. However, it turned out that *M. menardi* only rarely have the opportunity to catch prey during winter in caves. Starvation hardiness, along with exploiting any opportunity to catch prey, when available, appear as possible preadaptations to the subterranean habitat in this species. In this respect, the same evolutionary pathway can be expected in most orb-weaving spiders inhabiting subterranean habitats.

The significant differences in lipid droplet diameter, protein granule diameter and glycogen rosette counts in the midgut epithelial cells of *M. menardi* in winter, undergoing starvation under controlled and under natural conditions in caves, reveal that considerable differences in feeding conditions may occur among individuals. This was expected, since this is usual among spiders (e.g.^68^). On the other hand, the very similar courses of spending the three reserve compounds during winter starvation reveal stable physiological exploitation of the reserve compounds within the cells.

## Conclusions

We here draw conclusions on two issues: (1) Findings on ultrastructural changes in the midgut diverticula cells of *M. menardi*, wintering under natural conditions in caves (this study), and (2) Setting the theory on the key features making *M. menardi* a troglophile, based on previously compiled knowledge. This knowledge reveals many aspects of the biology and ecology of *M. menardi*, including its adaptation to a long-term deficiency of prey in the preferred habitat, like the twilight cave zone, in winter.

(1) We revealed that on the cellular level, in starved wintering *M. menardi*, changes appear in the midgut diverticula epithelial cells, typical of overwintering processes in many other arthropods. These are intensification of autophagy and spherite exploitation, along with gradual depletion of reserve lipids, glycogen and proteins. Thus, *M. menardi* is well adapted to survive natural winter starvation. This is a general survival pattern in many epigean arthropods under winter starvation, considered a possible preadaptation to the twilight zone of the natural subterranean habitat. We found no special features from a cytological perspective.

(2) Some specific biological, ecological, physiological and behavioural features are characteristic of *M. menardi*. They prefer the twilight zone in caves, in interspaces between stones in stone heaps and in similar subterranean habitats, where the temperature rarely falls below 0 °C, humidity remains relatively high and prey is abundant. They reproduce in the subterranean habitats only. In response to living there, *M. menardi* displays some general features characteristic of spiders, which we consider here possible preadaptations, and some special responses, unique or rarely met among the orb-weaving spiders. Although *M. menardi* can withstand well starvation, as most spiders do, they are active throughout the year and catch occasional prey whenever available. *Meta menardi* make a relatively small orb with a large mesh, which can ensnare mostly larger prey only, but combine this deficit with leaving the orb to capture prey on the bare walls. Additionally, *M. menardi* are in the process of diminishing tolerance for temperatures much below 0 °C, from moderate to minor tolerance.

Thus, *M. menardi* combines starvation hardiness and extremely opportunistic diet, both considered possible preadaptations, with some special features, like a partly reduced orb, tracking and capturing prey on the bare cave walls, and partly reduced tolerance to below-zero temperatures. All these make *M. menardi* well adapted to the transition, i.e. the twilight zone between the entrance and the deep cave zones. *Meta menardi* proves to be a model species to study adaptatiogenesis to the subterranean habitat in orb-weaving spiders.

## Material and Methods

For the study, we collected 10 males and 10 females from three caves (locality centroid 46°24′55″N, 15°10′31″E; altitude 600–740 m) in northern Slovenia at the beginning (November), in the middle (January) and at the end of wintering (March). We studied ultrastructural changes of the midgut epithelial cells in individuals spending winter under natural conditions in caves, using light microscopy and TEM.

### Light and transmission electron microscopy (TEM)

Small pieces of the midgut were fixed in 2.45% glutaraldehyde and 2.45% paraformaldehyde in a 0.1 M sodium cacodylate buffer (pH 7.4) at room temperature for 3 h, and at 4 °C for 14 h, washed in a 0.1 M sodium cacodylate buffer (pH 7.4) at room temperature for 3 h and postfixed with 2% OsO_4_ at room temperature for 2 h. The tissue was dehydrated in a graded series of ethanol (50%, 70%, 90%, 96%, 100%, each for 30 min at room temperature) and embedded in TAAB epoxy resin (Agar Scientific Ltd., Essex, England). For light microscopy, semi-thin sections (500 μm) of the midgut diverticula were stained with 0.5% toluidine blue in aqueous solution and analysed by a Nikon Eclipse E800 light microscope equipped with a Nikon DN100 camera. Ultra-thin sections (75 nm) were transferred onto copper grids, stained with uranyl acetate and lead citrate and analysed by a Zeiss EM 902 transmission electron microscope. For each sex and time frame, the percentage of epithelial cells with autophagic structures was calculated by random counting in 300 midgut epithelium cells. Autophagic structures were counted at the 3000x magnification. Cells containing autophagic structures were considered autophagic cells.

### Quantification of reserve lipids, glycogen and proteins by TEM

To estimate conditions with respect to these reserve compounds in the midgut epithelial cells during wintering, for each time frame and sex, we measured the diameter of 125 lipid droplets and 30 protein granules, and counted glycogen rosettes in 30 1-μm^2^ squares on the micrographs.

### Statistical analysis

The data distribution of lipid droplet diameter and protein granule diameter, and the glycogen rosette counts were tested for normality using the Kolmogorov-Smirnov test. The test showed a moderate difference in lipid droplets and glycogen rosettes; we therefore Log10-transformed the data for testing means. Two-way ANOVA was used for testing differences between means for sex, time frame and season. The t-test was used in testing differences between means under controlled and natural conditions.

### Ethical approval and informed consent

All the experiments were carried out in accordance with the relevant guidelines.

## Data Availability

The datasets generated during and/or analysed during the current study are available from the corresponding author on reasonable request.

## References

[1] Smithers P (2005). The diet of the cave spider *Meta menardi* (Latreille 1804) (Araneae, Tetragnathidae). The Joural of Arachnology.

[2] Novak T (2010). Niche partitioning in orbweaving spiders *Meta menardi* and *Metellina merianae* (Tetragnathidae). Acta Oecologica.

[3] Hörweg C, Blick T, Zaenker S (2011). Die Große Höhlenspinne *Meta menardi* (LATREILLE, 1804) – Höhlentier des Jahres und Europäische Spinne des Jahres 2012. Mitteilungen des Verbandes der deutschen Höhlen- und Karstforscher.

[4] Helsdingen, P. J. V. Araneae, IN: Fauna Europaea. Database European spiders and their distribution - Faunistics - Version 2017.1, http://www.european-arachnology.org/reports/fauna.shtml (2017).

[5] Mammola S, Isaia M (2014). Niche differentiation in *Meta bourneti* and *M. menardi* (Araneae, Tetragnathidae) with notes on the life history. International Journal of Speleology.

[6] Mammola, S. & Isaia, M. Rapid poleward distributional shifts in the European cave-dwelling *Meta* spiders under the influence of competition dynamics. *Journal of Biogeography*, 10.1111/jbi.13087, 1−9 (2017a).

[7] Mammola, S. & Isaia, M. Spiders in caves. *Proceedings of the Royal Society B: Biological Sciences* **284**, 10.1098/rspb.2017.0193 (2017b). 10.1098/rspb.2017.0193 PMC5413924 28446696

[8] Fritzén NR, Koponen S (2011). The cave spider *Meta menardi* (Araneae, Tetragnathidae) – occurrence in Finland and notes on its biology. Memoranda Societatis pro Fauna et Flora Fennica.

[9] Nentwig, W., Blick, T., Gloor, D., Hanggi, A. & Kropf, C. Spiders of Europe, Version 472 02.2017., www.araneae.unibe.ch (2017).

[10] Mammola S, Cardoso P, Ribera C, Pavlek M, Isaia M (2018). A synthesis on cave-dwelling spiders in Europe. Journal of Zoological Systematics and Evolutionary Research.

[11] Leruth R (1939). La biologie du domaine souterrain et la faune cavernicole de la Belgique. Mémoires du Museum d’histoire Naturelle de la Belgique.

[12] Tercafs R (1972). Biométrie spatiale dans l’écosystème souterraine: repartition du *Meta menardi* Latr. (Argiopidae). International Jounal of Speleology.

[13] Růžička V (1990). The spiders of stony debris. Acta Zoologica Fennica.

[14] Marusik YM, Koponen S (1992). A review of *Meta* (Araneae, Tetragnathidae), with description of two new species. Journal of Arachnology.

[15] Smithers P (1996). Observations on prey of the cave spider *Meta menardi* (Latreille 1804) in South Devon. Newsletter of the British Arachnological Society.

[16] Smithers P (2005). The early life history and dispersal of the cave spider *Meta menardi* (Latreille 1804), Tetragnathidae. Bulletin of the British Arachnological Society.

[17] Novak T, Perc M, Lipovšek S, Janžekovič F (2012). Duality of terrestrial subterranean fauna. International Journal of Speleology.

[18] Isaia, M. *et al*. Aracnidi sotterranei delle Alpi Occidentali italiane/Subterranean Arachnids of the Western Italian Alps (Arachnida: Araneae, Opiliones, Palpigradi, Pseudoscorpiones). Monografie XLVII. Museo Regionale di Scienze Naturali, Torino (2011).

[19] Manenti R, Lunghi E, Ficetola GF (2015). The distribution of cave twilight-zone spiders depends on microclimatic features and trophic supply. Invertebrate Biology.

[20] Legrand RS, Morse DH (2000). Factors driving extreme sexual size dimorphism of a sit-and-wait predator under low density. Biological Journal of the Linnean Society.

[21] Pötzsch JN (1966). zur Ernährung und Lebensweise von *Meta menardi* Latr. (Araneae; Araneidae). Abhandlungen des Berliner Naturkundemuseums.

[22] Bourne, J. D. & Robert, J. Remarques écologiques sur un population de l’aragnée troglophile *Meta menardi* Latreille. *Actes du 6*^*eme*^*Congr. suisse Spéléol*., Porrentruy, 25−35 (1978).

[23] Levi HW (1980). The orb-weaver genus *Mecynogea*, the subfamily Metinae and the genera *Pachygnatha*, *Glenognatha* and *Azilia* of the subfamily Tetragnathinae north of Mexico (Araneae: Araneidae). Bulletin of the Museum of Comparative Zoology.

[24] Eckert R, Moritz M (1992). *Meta menardi* (Latr.) and *Meta merianae* (Scop.): On the biology and habitat of the two commonest spiders in the caves of the Harz, the Kyffhauser, Thuringia and the Zittau Mountains. Mitteilungen aus dem Zoologischen Museum in Berlin.

[25] Szymczakowski W (1953). Preferendum temniczne jaskiniowego paja, ka “*Meta menardi*” Latr. (Argiopidae). Folia Biologica.

[26] Dresco-Derouet L (1960). Étude biologique comparé de quelques espèces d’araignées lucicoles et troglophiles. Archive de Zoologie Experimentale et Générale.

[27] Tercafs R (1960). Notes à propos de deux araignées cavernicoles “*Meta menardi* Latr.” et “*Nesticus cellulanus* Clerck (Argiopidae)”. Annales de la Féderation de Spéléologie Belgique.

[28] Bourne JD (1976). Notes préliminaires sur la distribution spatiale de *Meta menardi*, *Triphosa dubitata*, *Triphosa sabaudiata*, *Nelima aurantiaca* et *Culex pipiens* au sain d’un écosystème cavernicole (Grotte de Scierie: Mte.-Savoie). International Journal of Speleology.

[29] Bourne JD (1977). Mise en evidence de groupements temporaires de la faune pariétale dans un tunnel artificiel en fonction de l’humidité et des mouvements d’air. Revue Suisse de Zoology.

[30] Nyffeler M, Symondson WOC (2001). Spiders and harvestmen as gastropod predators. Ecological Entomology.

[31] Lepore E, Marchioro A, Isaia M, Buehler MJ, Pugno NM (2012). Evidence of the most stretchable egg sac silk stalk, of the European spider of the year *Meta menardi*. PLoS ONE.

[32] Chiavazzo E (2015). Cave spiders choose optimal environmental factors with respect to the generated entropy when laying their cocoon. Scientific Reports.

[33] Lipovšek S (2017). Changes in the midgut cells in the European cave spider, *Meta menardi*, during starvation in spring and autumn. Histochemistry and Cell Biology.

[34] Lunghi E, Manenti R, Ficetola GF (2017). Cave features, seasonality and subterranean distribution of non-obligate cave dwellers. PeerJ.

[35] Schiner, J. R. Fauna der Adelsberger-, Lueggerund Magdalenen-Grotte. In: Schmidl, A. ed. *Die Grotten und Höhlen von Adelsberg, Lueg, Planina und Laas*. 231−272 (Wien: Braumüller, 1854).

[36] Racoviță EG (1907). Essai sur les problemes biospéologiques. Archives de Zoologie Expérimentale et Générale (Biospéologica I), 4e serie.

[37] Mammola S (2018). Finding answers in the dark: caves as models in ecology fifty years after Poulson and White. Ecography.

[38] Lunghi E, Manenti R, Ficetola GF (2014). Do cave features affect underground habitat exploitation by non-troglobite species?. Acta Oecologica.

[39] Sket B (2008). Can we agree on an ecological classification of subterranean animals?. Journal of Natural History.

[40] Culver, D. C. & Pipan, T. *The Biology of Caves and Other Subterranean Habitats*. 256 p. (Oxford University Press, Oxford, New York, 2009).

[41] Kirchner, W. Behavioural and physiological adaptations to cold. In: Nentwig, W. ed. *Ecophysiology of Spiders*. Berlin: Springer-Verlag, 66–77 (1987).

[42] Novak T (2014). Cold tolerance in terrestrial invertebrates inhabiting subterranean habitats. International Journal of Speleology.

[43] Lipovšek S, Novak T, Janažekovič F, Brdelak N, Leitinger G (2018). Changes in the midgut diverticula epithelial cells of the European cave spider, *Meta menardi*, under controlled winter starvation. Scientific Reports.

[44] Nawabi, S. *Histologische Untersuchungen an der Mitteldarmdrüse von Stegodyphus pacificus* (Pocock 1900) (Araneae, Eresidae). M.Sc, thesis, Univ. Bonn, Germany (1974).

[45] Lipovšek S, Janžekovič F, Novak T (2017). Ultrastructure of fat body cells and Malpighian tubule cells in overwintering *Scoliopteryx libatrix* (Noctuoidea). Protoplasma.

[46] Foelix, R. F. *Biology of Spiders*. (New York: Oxford University Press, 1996).

[47] Felgenhauer, B. E. Araneae. In: Harrison, F. W. & Foelix, R. F. eds *Microscopic Anatomy of Invertebrates*. *Volume 8A**:**Chelicerate Arthropoda*. 223−266 (New York: Wiley-Liss, 1999).

[48] Mizushima N, Ohsumi Y, Yoshimori T (2002). Autophagosome formation in mammalian cells. Cell Structure and Function.

[49] Xie Z, Klionsky DJ (2007). Autophagosome formation: core machinery and adaptations. Nature Cell Biology.

[50] Lipovšek S, Novak T (2016). Autophagy in the fat body cells of the cave cricket *Troglophilus neglectus* Krauss, 1878 (Rhaphidophoridae, Saltatoria) during overwintering. Protoplasma.

[51] Lipovšek S, Novak T, Janžekovič F, Senčič L, Pabst MA (2004). A contribution to the functional morphology of the midgut gland in phalangiid harvestmen *Gyas annulatus* and *Gyas titanus* during their life cycle. Tissue & Cell.

[52] Lipovšek, S., Novak, T., Janžekovič, F. & Leitinger, G. Changes in the midgut diverticula in the harvestmen *Amilenus aurantiacus* (Phalangiidae, Opiliones) during winter diapause. *Arthropod Structure & Development*., 10.1016/j.asd.2014.12.002 (2015). 10.1016/j.asd.2014.12.002 25546311

[53] Belozerov VN (2008). Diapause and quiescence as two main kinds of dormancy and their significance in life cycles of mites and ticks (Chelicerata: Acarina: Acari). Part 1. Acariformes. Acarina.

[54] Haeler E, Fiedler K, Grill A (2014). What prolongs a butterfly’s life? Trade-offs between dormancy, fecundity and body size. PLoS ONE.

[55] Diniz DFA, Ribeiro de Albuquerque CM, Oliva LO, de Melo-Santos MAV, Ayres CFJ (2017). Diapause and quiescence: dormancy mechanisms that contribute to the geographical expansion of mosquitoes and their evolutionary success. Parasites & Vectors.

[56] Edgar WE, Loenen M (1974). Aspects of the overwintering habitat of adult females of the wolf spider *Pardosa amentata* (Clerck). Journal of Zoology (London).

[57] Mammola, S., Piano, E. & Isaia, M. Step back! Niche dynamics in cave-dwelling predators. Acta Oecologica 75: 35–42, 10.1016/j.actao.2016.06.011(2016).

[58] Pulliam HR (1988). Sources, sinks, and population regulation. American Naturalist.

[59] Trajano E, de Carvalho MR (2017). Towards a biologically meaningful classification of subterranean organisms: a critical analysis of the Schiner-Racovitza system from a historical perspective, difficulties of its application and implications for conservation. Subterranean Biology.

[60] Wilczek G (2014). Apoptotic and necrotic changes in the midgut glands of the wolf spider *Xerolycosa nemoralis* (Lycosidae) in response to starvation and dimethoate exposure. Ecotoxicology and Environmental Safety.

[61] Ludwig M, Alberti G (1990). Peculiarities of arachnid midgut glands. Acta Zoologica Fennica.

[62] Lipovšek S, Janžekovič F, Novak T (2014). Autophagic activity in the midgut gland of the overwintering harvestmen *Gyas annulatus* (Phalangiidae, Opiliones). Arthropod Structure & Development.

[63] Pigino G, Migliorini M, Paccagnini E, Bernini F, Leonzio C (2005). Fine structure of the midgut and Malpighian papillae in *Campodea* (*Monocampa*) *quilisi* Silvestri, 1932 (Hexapoda, Diplura) with special reference to the metal composition and physiological significance of midgut intracellular electron-dense granules. Tissue and Cell.

[64] Arrese EL (2001). Lipid storage and mobilization in insects: current status and future directions. Insect Biochemistry and Molecular Biology.

[65] Arrese EL, Soulages JL (2010). Insect fat body: energy, metabolism, and regulation. Annual Review in Entomology.

[66] Hahn DA, Denlinger DL (2011). Energetics of insect diapause. Annual Review in Entomology.

[67] Laino A, Cunningham ML, García F, Heras H (2009). First insight into the lipid uptake, storage and mobilization in arachnids: Role of midgut diverticula and lipoproteins. Journal of Insect Physiology.

[68] DiRienzo N, Montiglio P-O (2016). Linking consistent individual differences in web structure and behavior in black widow spiders. Behavioral Ecology.

